# Gd-based Contrast Enhancement of the Perivascular Spaces in the Basal Ganglia

**DOI:** 10.2463/mrms.mp.2016-0039

**Published:** 2016-07-12

**Authors:** Shinji Naganawa, Toshiki Nakane, Hisashi Kawai, Toshiaki Taoka

**Affiliations:** Department of Radiology, Nagoya University Graduate School of Medicine 65 Tsurumai-cho, Shouwa-ku, Nagoya, Aichi 466-8550, Japan

**Keywords:** magnetic resonance imaging, perivascular space, gadolinium, glymphatic system

## Abstract

**Purpose:**

In textbooks, the perivascular space (PVS) is described as non-enhancing after the intravenous administration of gadolinium-based contrast agent (IV-GBCA). We noticed that the PVS sometimes has high signal intensity (SI) on heavily T_2_-weighted 3D-FLAIR (hT_2_-FL) images obtained 4 h after IV-GBCA. The purpose of this study was to retrospectively evaluate the contrast enhancement of the PVS.

**Materials and Methods:**

In 8 healthy subjects and 19 patients with suspected endolymphatic hydrops, magnetic resonance cisternography (MRC) and hT_2_-FL images were obtained before and 4 h after a single dose of IV-GBCA. No subjects had renal insufficiency. On axial MRC at the level of the anterior commissure (AC)-posterior commissure (PC) line, 1 cm circular regions of interest (ROIs) were drawn centering on the PVS in the bilateral basal ganglia and thalami. Three-millimeter diameter ROIs were set in the cerebrospinal fluid (CSF) of the bilateral ambient cistern. The ROIs on MRC were copied onto the hT_2_-FL images and the SI was measured. The SI ratio (SIR) was defined as SIR_PVS_ = SI of PVS/SI of the thalami, and SIR_CSF_ = SI of CSF/SI of the thalami. The average of the bilateral values was used for the calculation. The SIR_CSF_, SIR_PVS_, and SI of the thalami were compared between before and 4 h after IV-GBCA.

**Results:**

The SIR was increased significantly from 1.02 ± 0.37 to 2.65 ± 0.82 in the CSF (P < 0.01) and from 1.20 ± 0.35 to 2.13 ± 1.23 in the PVS at 4 h after IV-GBCA (*P* < 0.01). The SI of the thalami showed no significant difference.

**Conclusion:**

The enhancement of the PVS at 4 h after IV-GBCA was confirmed even in subjects without renal insufficiency. It is possible that the GBCA in the blood vessels might have permeated into the cerebrospinal fluid (CSF) space and the PVS. This might be a first step in the imaging evaluation of the glymphatic system (waste clearance system) of the brain.

## Introduction

In textbooks, the perivascular space (PVS) is described as non-enhancing after the intravenous administration of gadolinium-based contrast agent (IV-GBCA).^1^ The PVS exists throughout the brain, but is most frequently seen in the inferior third of the basal ganglia near the anterior commissure (AC).^1^ We routinely performed magnetic resonance (MR) imaging for the assessment of endolymphatic hydrops 4 h after a single dose of IV-GBCA.^2–7^ We noticed occasionally that the PVS would have high signal intensity (SI) on heavily T_2_-weighted 3D-FLAIR (hT_2_-FL) images obtained 4 h after a single dose of IV-GBCA. hT_2_-FL imaging has been utilized as a sensitive technique to detect very low concentrations of GBCA in fluid.^4,8,9^ The slight enhancement of the cerebrospinal fluid (CSF) on hT_2_-FL images acquired 4 h after a single dose of IV-GBCA had been reported in healthy subjects with normal renal function^10^; however, the enhancement of the PVS in healthy subjects has not been reported previously. The enhancement of the PVS, though, has been reported in a patient with renal insufficiency.^11^ The PVS represents an entrance point to the glymphatic system, a recently discovered macroscopic waste clearance system of the brain.^12,13^

The purpose of this study was to retrospectively evaluate the contrast enhancement of the PVS both prior to and 4 h after a single dose of IV-GBCA in subjects who underwent MR imaging.

## Materials and Methods

In 8 healthy men (ages: 29–53) and 19 patients with suspected endolymphatic hydrops (7 men, 12 women; ages: 27–75), MR cisternography (MRC), and hT_2_-FL images were obtained on a 3T scanner (Magnetom Verio, Siemens Healthcare, Erlangen, Germany) prior to and 4 h after a single dose of IV-GBCA. No subjects had renal insufficiency. The 8 volunteers and 7 of the patients received IV-GBCA of 0.1 mmol/kg body weight or 0.2 ml/kg body weight using Gadoteridol (Gd-HP-DO3A: ProHance, Eisai, Tokyo, Japan) and the other 12 patients received the same amount of IV-GBCA using Gadodiamide (Gd-DTPA-BMA: Omniscan, Daiichi-Sankyo, Tokyo, Japan). In this study, we defined renal insufficiency as the history of proteinuria within 3 months or the estimated glomerular filtration rate (eGFR) <60 mL/min/1.73 m^2^.

All MR scans were performed using 32-channel array head coil and GRAPPA (acceleration factor of 2). The slice thickness, field of view, matrix size, and slice position were identical in both the MRC and hT_2_-FL images. The voxel size was 0.5 × 0.5 × 1.0 mm^3^. All sequences utilize a frequency selective fat suppression pre-pulse. Further details of the MR parameters are listed in Table 1. On the axial MRC parallel to the AC-posterior commissure (PC) line, a 1 cm circular region of interest (ROI) was drawn and centered on the PVS bilaterally in both the basal ganglia and in the center of the thalami for signal reference.

**Fig 1.**
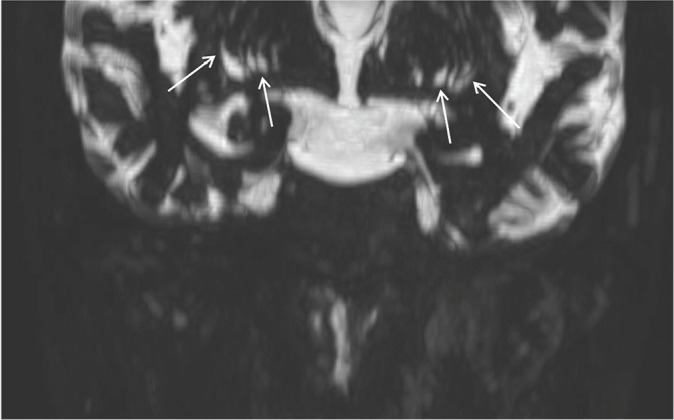
Coronal reformatting is performed to differentiate a large perivascular space (PVS) from the cerebrospinal fluid (CSF)-containing lacunes. On a coronal reformatted image, the PVS (arrows) shows a typical shape following perforating vessels without surrounding gliosis.

The PVS was defined as “small, sharply delineated structures with CSF-like intensity on the MRC that followed the course of the perforating arteries.” If a CSF-like signal area of 3 mm or above was found, coronal reformatting was performed to exclude the CSF containing lacunes by checking the shape (Fig. 1).^14^

The circular 1 cm diameter ROI for the PVS in basal ganglia was placed according to the instructions given below.

**Fig 2.**
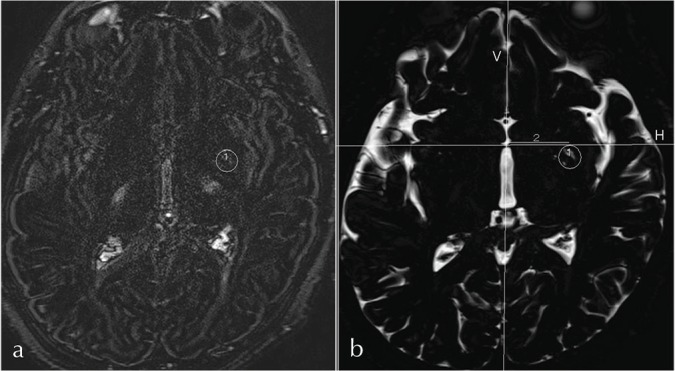
Example of region of interest (ROI) placement. On magnetic resonance cisternography (MRC) (**a**) at the anterior commissure (AC)-posterior commissure (PC) slice, a horizontal line (line H) was drawn through the AC vertical to the midline (line V). A 1 cm diameter ROI was drawn just posterior to line H (circle 1). The distance from midline (length of line 2) was 2.5–3 cm. The ROI was placed to include as much of the perivascular space (PVS) as possible. If the PVS is not clear on MRC, line 2 was placed at 2.75 cm. Then, the ROI was copied onto the hT_2_-FL image (**b**).

“Select the highest axial MRC slice that contains both the AC and PC. Draw the vertical line along the center of the interhemispheric fissure (line V on Fig. 2b). Draw the horizontal line perpendicular to line V through the AC (line H on Fig. 2b). Draw an ROI (ROI 1 on Fig. 2) posterior to line H contacting the anterior edge of the circle. The distance between the contacting point and midline (Line 2 on Fig. 2b) should be 25–30 mm. The ROI should be placed to cover as many PVS as possible on the MRC. If the VRS is not clear on the MRC, a distance of 27.5 mm should be employed.” Then, the ROIs were copied onto the hT_2_-FL image and the SI of the ROI was measured (Fig. 2b).

The circular ROIs of 1 cm diameter were placed in the bilateral thalami on the same slice as the PVS at 1 cm lateral to the midline. The 3 mm diameter ROIs were set within the bilateral CSF spaces of the ambient cistern, avoiding the vessels. Then, the ROIs were copied onto the hT_2_-FL image and the SI of the ROI was measured. We chose the ambient cistern for the signal measurement of CSF according to the previous study.^10^ In ambient cistern, the apparent signal decrease of CSF due to flow was not seen on MRC, and the significant contrast enhancement was detected on HF in the previous volunteers’ study. The significant enhancement in lateral ventricle was not detected in that study.^10^ One experienced neuroradiologist placed the ROIs on the MRC.

The SI ratio (SIR) was defined as: SIR_PVS_ = SI of PVS/SI of the thalami, and SIR_CSF_ = SI of CSF/SI of the thalami. The average of the bilateral values was used for the calculation. The signal enhancement ratio (SER) of the PVS was given as SER = SIR of the post-contrast administration/SIR of the pre-contrast administration.

The SIR_CSF_, SIR_PVS_, and SI of the thalami were compared prior to and 4 h post IV-GBCA using Student’s *t*-test. The SER of the volunteers and patients were compared using Mann-Whitney’s test. We set 5% as a significance level for the statistical test.

For the healthy volunteers’ scans, the medical ethics committee of our institution approved the study and written informed consent was obtained from all volunteers.

For the patients’ retrospective study, the medical ethics committee of our institution approved this retrospective study with a waiver of written informed consent from the patients.

## Results

**Fig 3.**
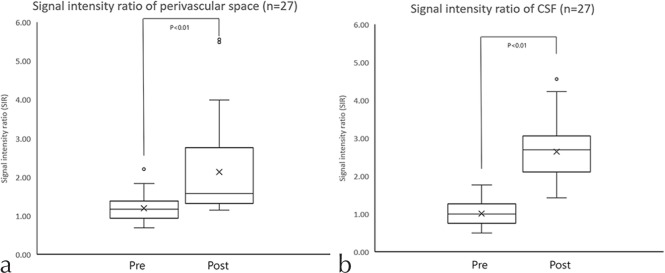
The signal intensity ratio (SIR) distribution in all 27 subjects. (**a**) The SIR of the perivascular space was increased significantly at 4 h after intravenous administration of gadolinium-based contrast agent (IV-GBCA). (**b**) The SIR of the cerebrospinal fluid (CSF) was increased significantly at 4 h after IV-GBCA.

**Fig 4.**
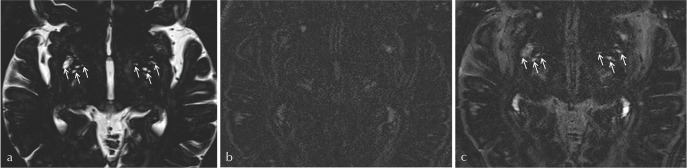
A 71-year-old woman with suspected endolymphatic hydrops. On magnetic resonance cisternography (MRC) (**a**), there is much perivascular space (PVS) in the bilateral basal ganglia (arrows). On pre-contrast heavily T_2_-weighted FLAIR (hT_2_-FL) image, the PVS has a low-signal intensity (**b**). On post-contrast hT_2_-FL images (**c**), the PVS has an increased signal intensity (arrows). The cerebrospinal fluid (CSF) also has increased signal. Note that some of the PVS has a higher signal than the CSF.

In all 27 subjects, the mean SIR_CSF_ increased significantly from 1.02 ± 0.37 to 2.65 ± 0.82 after the IV-GBCA administration (*P* < 0.01) and the mean SIR_PVS_ increased significantly from 1.20 ± 0.35 to 2.13 ± 1.23 after IV-GBCA (*P* < 0.01) (Figs. 3, 4).

No significant difference was found between the SI of the thalami prior to and 4 h after IV-GBCA.

When limited to the 8 volunteers and 7 patients who received IV-GBCA by Gadoteridol, the mean SIR_CSF_ increased significantly from 0.91 ± 0.32 to 2.35 ± 0.72 after the IV-GBCA administration (*P* < 0.01) and the mean SIR_PVS_ increased significantly from 1.00 ± 0.16 to 1.38 ± 0.14 after IV-GBCA (*P* < 0.01).

When limited to the 12 patients who received IV-GBCA by Gadodiamide, the mean SIR_CSF_ increased significantly from 1.15 ± 0.11 to 3.03 ± 0.22 after the IV-GBCA administration (*P* < 0.01) and the mean SIR_PVS_ increased significantly from 1.46 ± 0.10 to 3.07 ± 0.37 after IV-GBCA (*P* < 0.01).

The SER of the PVS and the SER of the CSF showed no significant difference between the 8 volunteers and 19 patients. When limited to the Gadoteridol group, the SER of the PVS and the SER of the CSF showed no significant difference between the 8 volunteers and 7 patients. Among the patients who received IV-GBCA by Gadodiamide and that by Gadoteridol, there was no significant difference in the SER of the PVS or the SER of the CSF.

## Discussion

In this study, the enhancement of the PVS and CSF was confirmed even in subjects without renal insufficiency. The high sensitivity of the hT_2_-FL image to a low concentration of GBCA in fluid allowed the detection of the slightest enhancement of the PVS in subjects without renal insufficiency.^4,8^ It has been reported that GBCA injected intrathecally enters into the PVS and brain parenchyma.^15^ In this study, it is speculated that the IV-GBCA entered into the PVS from the CSF. In the previous healthy volunteer study, the timing of enhancement is earlier in the optic nerve sheath, Meckel’s cave around trigeminal nerve, and the fundus of the internal auditory canal around the seventh and eighth cranial nerves than in the CSF of the ambient cistern.^10^ These findings suggested that GBCM permeated from the peripheral part of the cranial nerve or nerve sheath to CSF, even in the subjects with intact blood–brain barrier.

On the other hand, some of the PVS signals were visually higher than that of the CSF at 4 h after IV-GBCA (Fig. 4). Simple penetration of the CSF into the interstitial fluid of the PVS cannot explain this more intensely increased PVS signal. This suggests that the PVS has an active mechanism such as the absorption of water in the PVS or the secretion of gadolinium into the PVS. This active function of the PVS might be investigated using MR imaging in the future. A signal decrease of CSF due to flow cannot be completely ruled out; however, the CSF signal of ambient cistern on MRC was quite high as inner ear fluid. Therefore, the flow effect in ambient cistern might be quite small. Most reports regarding the glymphatic system are describing the PVS around artery at the entry site of CSF to the glymphatic system^12^; however, one report is describing the PVS around the artery as both the entry and outlet.^16^ If the PVS around perforating artery acts also as an outlet, more intense enhancement of PVS than CSF seems to be reasonable. The MR scans at most time points should be performed in the future to reveal the precise kinetics of GBCA in the brain. The PVS in another region of the brain should also be investigated. The glymphatic system is reported to be active during sleep^17^; thus, the sleep condition should be controlled during the investigation of the glymphatic system.

The findings of this study suggest that the blood–CSF barrier and the CSF–interstitial fluid (IF) barrier might be leakier than the blood–brain barrier. The deposition of gadolinium in the brain parenchyma such as in the dentate nucleus and globus pallidus is a recent hot topic in the field of MR imaging.^18,19^ Intact gadolinium-chelates might penetrate the brain parenchyma through the CSF and PVS (i.e., the glymphatic system).

Recently, several reports have suggested that the PVS is a part of the brain “lymphatic” system (glymphatic system) through which the interstitial solutes are cleared from the brain^12,13,20,21^ It has been demonstrated that arterial pulsation drives the subarachnoid CSF flow into the PVS, clearing soluble proteins such as amyloid beta (Aβ) from the brain.^20,21^ The wastes are washed out of brain from peri-venous spaces to CSF space. Dysfunction of the PVS pathways thus may lead to an enlarged PVS, increased Aβ deposition, and subsequent neuronal dysfunction and loss, which clearly would have profound implications for Alzheimer’s disease.^21^

In the animal study, an intrathecal injection of gadolinium was utilized to investigate glymphatic function by analyzing the CSF-ISF exchange across the brain.^20^ The results of this study might open the door to an examination of the glymphatic system in humans by a clinically feasible method using MR imaging and IV-GBCA.

## Conclusion

The enhancement of the PVS at 4 h after a single dose of IV-GBCA was confirmed in human subjects without renal insufficiency. This might represent the first steps using MR imaging to evaluate the glymphatic system in humans.

## Figures and Tables

**Table 1.** Pulse sequence parameters

Sequence name	Type	Repetition time (ms)	Echo time (ms)	Inversion time (ms)	Flip angle (degree)	Section thickness/gap (mm)	Pixel size (mm)	Number of slices	Echo train length	Field of view (mm)	Matrix size	Number of excitations	Scan time (min:s)
MR cisternography	SPACE with restore pulse	4400	544	NA	90/initial 180 decrease to constant 120	1/0	0.5 × 0.5	104	173	165 × 196	324 × 384	1.8	3:13
Heavily T_2_-weighted 3D-FLAIR	SPACE with inversion pulse	9000	544	2250	90/initial 180 decrease to constant 120	1/0	0.5 × 0.5	104	173	165 × 196	324 × 384	2	7:21

MR, magenetic resonance; NA, not applied; SPACE, sampling perfection with application-optimized contrasts using different flip angle evolutions
